# Laboratory biomarkers and immunological modulation of sintilimab in gastric cancer: A meta-analysis focused on tumor markers and T-cell subsets

**DOI:** 10.5937/jomb0-59513

**Published:** 2025-09-05

**Authors:** Xian Zhang, Qiang Zhao, Huafei Tang, Rui Qin, Ting Tian, Congying Li, Rui Ma

**Affiliations:** 1 The 305 Hospital of PLA, Department of Pharmacy, Beijing, China; 2 The 305 Hospital of PLA, Department of Gastroenterology, Beijing, China; 3 Capital Medical University Electric Power Teaching Hospital (State Grid Corporation of China Beijing Electric Power Hospital), Department of Cardiology, Beijing, China; 4 Beijing Rehabilitation Hospital, Capital Medical University, Department of Pharmacy, Beijing, China

**Keywords:** sintilimab, gastric cancer, CEA, CA199, CA242, CD4+, CD8+, biochemical markers, laboratory diagnostics, meta-analysis, sintilimab, rak želuca, CEA, CA199, CA242, CD4+, CD8+, biohemijski markeri, laboratorijska dijagnostika, meta-analiza

## Abstract

**Background:**

Sintilimab, a PD-1 inhibitor, has emerged as a promising immunotherapeutic agent in gastric cancer. However, its impact on laboratory-based biochemical markers and immune indicators remains underexplored. This meta-analysis aimed to evaluate the changes in tumor biomarkers and T lymphocyte subsets, alongside clinical outcomes, in patients receiving sintilimab.

**Methods:**

A comprehensive literature search of randomized controlled trials (2022-2025) was conducted across CNKI, Wanfang, VIP, and PubMed databases. Primary outcomes included serum tumor markers (CEA, CA199, CA242) and immune parameters (CD4+, CD8+ T-cell subsets). Secondary outcomes were ORR, DCR, OS, PFS, and adverse reactions. RevMan 5.2 was used for meta-analysis.

**Results:**

Sixteen studies were included. Sintilimab treatment significantly reduced CEA, CA199, and CA242 levels (P < 0.0001), and favorably modulated immune subsets by increasing CD4+ and decreasing CD8+ cell counts. These biochemical and immunological improvements correlated with higher ORR, DCR, and OS, without increased adverse events (P > 0.05).

**Conclusions:**

Sintilimab confers measurable improvements in key laboratory-based tumor and immune biomarkers, supporting its utility in clinical biochemical monitoring and immunotherapy response evaluation for gastric cancer patients. These findings align with the emerging integration of immunotherapy and biochemical diagnostics in oncology.

## Abstract (sr)

**Uvod:**

Sintilimab, inhibitor PD-1, pojavio se kao obećavajući imunoterapeutski agens kod raka želuca. Međutim, njegov uticaj na laboratorijske biohemijske markere i imunološke indikatore ostaje nedovoljno istražen. Cilj ove meta-analiza bio je da se procene promene u tumorskim biomarkerima i podskupovima T limfocita, zajedno sa kliničkim ishodima, kod pacijenata koji primaju sintilimab.

**Metode:**

Sprovedena je sveobuhvatna pretraga literature randomizovanih kontrolisanih studija (2022-2025) u bazama podataka CNKI, Wanfang, VIP i PubMed. Primarni ishodi obuhvatali su serumske tumorske markere (CEA, CA199, CA242) i imunološke parametre (CD4+, CD8+ T-ćelijske podgrupe). Sekundarni ishodi bili su ORR, DCR, OS, PFS i neželjene reakcije. Za meta-analizu je korišćen RevMan 5.2.

**Rezultati:**

Uključeno je šesnaest studija. Lečenje sintilimabom značajno je smanjilo nivoe CEA, CA199 i CA242 (P < 0,0001) i povoljno moduliralo imunološke podgrupe pove ćanjem broja CD4+ i smanjenjem broja CD8+ ćelija. Ova biohemijska i imunološka poboljšanja korelirala su sa višim ORR, DCR i OS, bez povećanja neželjenih događaja (P > 0,05).

**Zaključak:**

Sintilimab pruža merljiva poboljšanja ključnih la - boratorijskih tumorskih i imunoloških biomarkera, što podržava njegovu korisnost u kliničkom biohemijskom praćenju i proceni odgovora na imunoterapiju kod pacijenata sa rakom želuca. Ovi nalazi su u skladu sa novom integracijom imunoterapije i biohemijske dijagnostike u onkologiji.

## Introduction

Gastric cancer is a common malignant tumor of the digestive tract in clinical practice. Globally, its incidence ranks among the top five, and its mortality ranks among the top four [1] [2]. Early-stage gastric cancer is often treated with radical surgery. However, at present, there is a lack of effective and simple screening methods for gastric cancer, so most patients are diagnosed at an advanced stage, when the effectiveness of surgical treatment cannot meet expectations. Therefore, neoadjuvant therapies such as platinum combined with taxanes and fluorouracil can effectively improve clinical symptoms, and their efficacy has been confirmed, but using these regimens alone cannot achieve ideal therapeutic goals [3].

In recent years, programmed cell death protein 1 (PD-1) inhibitors have been shown to play a positive role in suppressing immune inhibition and activating T cells by blocking the binding of PD-1 and its ligand, thereby killing tumor cells and exerting anti-tumor effects [4] [5]. At present, the efficacy and safety of sintilimab in patients with gastric cancer are still being explored in depth, and conclusions remain inconsistent. Therefore, this study aims to use meta-analysis to investigate the efficacy, tumor markers, T lymphocyte subsets, and safety of sintilimab in the treatment of gastric cancer, providing data support for the future application and promotion of sintilimab regimens in patients with gastric cancer.

## Materials and methods

### Literature search

This meta-analysis focused on randomized controlled trials (RCTs) that investigated the biochemical and immunological effects of sintilimab in patients with gastric cancer. A comprehensive literature search was performed across both domestic and international databases, including VIP, CNKI, Wanfang Medical, and PubMed. The search employed keywords such as »sintilimab,« »efficacy,« »tumor markers,« »T lymphocyte subsets,« and »adverse reactions.« The publication time frame was restricted to the past five years (2022–2025) to ensure contemporary relevance.

To maximize the accuracy and integrity of the data, a systematic protocol based on pre-defined inclusion criteria and keyword matching was applied. Additionally, consultation with experienced biomedical researchers was undertaken to refine the search strategy and optimize the methodological approach. When necessary, corresponding authors were contacted to clarify unclear results or to obtain missing biochemical and laboratory data, particularly for outcome indicators such as CEA, CA199, CA242, and T-cell subset levels.

The scientific validity of each study was ensured through critical appraisal. Only studies that provided appropriate institutional approvals and ethical clearances were retained. Articles were excluded if they exhibited methodological flaws such as inconsistent outcome definitions or obvious data duplication. Ultimately, data extraction and synthesis were performed using RevMan 5.2 software for meta-analysis.

### Literature inclusion and exclusion criteria

Studies were included in this meta-analysis if they fulfilled the following criteria: being randomized controlled trials published from 2022 onward that compared the efficacy of sintilimab combined with chemotherapy to chemotherapy alone in patients diagnosed with gastric cancer; including participants of any age, sex, or ethnic background; and providing clear documentation of ethical approval and informed consent. To align with the journal’s focus on laboratory medicine, eligible studies were also required to report at least one quantifiable laboratory-based biochemical or immunological marker, such as serum tumor biomarkers (e.g., CEA, CA199, CA242) or T-cell subset levels (e.g., CD4 , CD8 ). Additionally, baseline characteristics between groups needed to be sufficiently balanced following randomization (except for sample size), and the rate of loss to follow-up had to be less than 10%.

Exclusion criteria were applied to ensure methodological rigor and relevance to the scope of the review. Specifically, non-original articles—including systematic reviews, case reports, meta-analyses, and conference abstracts—were excluded. Preclinical investigations involving animal models or in vitro cell lines were not considered, as the analysis was confined to human clinical data. Studies that failed to report laboratory-based biochemical outcomes or those not directly evaluating the combination of sintilimab with chemotherapy for gastric cancer were also excluded.

### Outcome indicators

Primary outcome indicators were centered on laboratory and biochemical measurements. These included serum tumor biomarkers—CEA, carbohydrate antigen 199 (CA199), and carbohydrate antigen 242 (CA242)—which are widely utilized in clinical laboratories for cancer detection, monitoring, and prognosis. Additionally, immune-related laboratory parameters such as CD4 and CD8 T lymphocyte subsets, commonly assessed via flow cytometry, were evaluated to reflect immune function modulation.

Secondary outcomes comprised clinical efficacy indicators: objective response rate (ORR), disease control rate (DCR), overall survival (OS), and progression- free survival (PFS). Safety was assessed by the incidence of common adverse events monitored in routine laboratory settings, including hematologic toxicity (e.g., myelosuppression), hepatotoxicity, gastrointestinal effects (e.g., nausea, vomiting), and neurotoxicity (e.g., peripheral sensory neuropathy).

### Quality assessment

The methodological quality of included trials was evaluated using the modified Jadad scale. This tool assesses randomization, blinding, and reporting quality, with scores ranging from 1 to 7. Studies scoring ≥4 were categorized as high quality, while those scoring 3 were classified as low quality.

### Statistical methods

Statistical analyses were conducted using Review Manager (RevMan) 5.2 software. For dichotomous outcomes, risk ratios (RRs) with 95% confidence intervals (CIs) were calculated. Continuous laboratory-based outcomes, such as serum biomarker levels and immune cell percentages, were analyzed using weighted mean differences (WMDs) or standardized mean differences (SMDs), depending on units and consistency of reporting. Heterogeneity among studies was assessed using the Chi-square test and I^2^ statistic. A random-effects model was used when I^2^ ≥ 50% or P < 0.1, indicating significant heterogeneity; otherwise, a fixed-effects model was applied.

## Results

### Literature search results and characteristics

**Figure 1:**
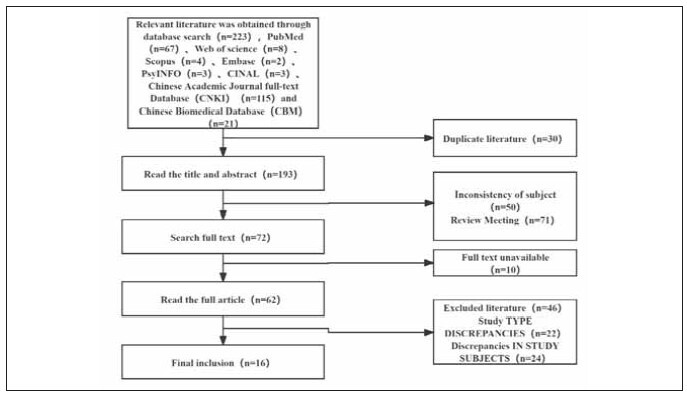
Flowchart of literature selection.

**Figure 2:**
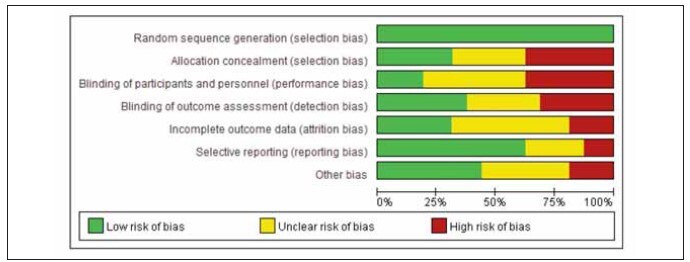
Overall publication bias plot of the included literature.

**Figure 3:**
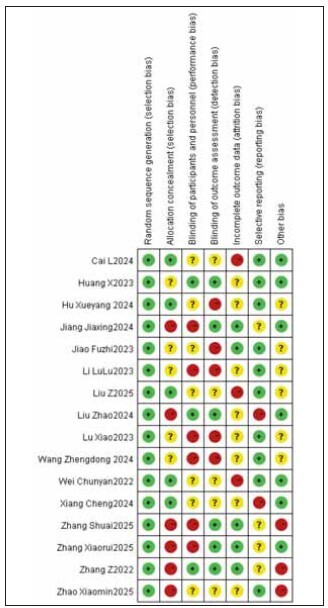
Publication bias plot of individual studies.

A total of 223 relevant articles were retrieved from Chinese and English databases including Wanfang, CNKI, and PubMed, according to the study’s main direction, keywords, and selection criteria. After screening according to the inclusion criteria, 12 Chinese articles and 4 English articles were included [6] [7] [8] [9] [10] [11] [12] [13] [14] [15] [16] [17] [18] [19] [20] [21]. The specific literature search process is shown in Figure 1. Among the included studies, 12 were high quality and 4 were low quality (Table 1). No significant publication bias was found among the 16 included studies (Figure 2, Figure 3).

**Table 1:** Basic characteristics of the literature. Note: ① ORR; ②DCR; ③ CA242; ④ CA199; ⑤ CEA; ⑥ CD4 ; ⑦ OS; ⑧ PFS; ⑨ Myelosuppression; ⑩ Nausea and vomiting; ⑪ Liver function impairment; ⑫ Peripheral sensory neuropathy; ⑬ CD8^+^.

Author	Year	Treatment Regimen<br>(Observation/Control)	Sample Size<br>(Obs/Con)	Outcome<br>Indicators	Quality<br>Score
Cai L [6]	2024	Sintilimab + control / Docetaxel<br>+ Cisplatin + 5-Fu	48/46	①②③④⑤	5
Liu Z [7]	2025	Sintilimab + control / Nab-paclitaxel +<br>S-1 + Trastuzumab	46/57	①②	3
Zhang Z [8]	2022	Sintilimab + control / Nab-paclitaxel	60/60	①⑦⑧	4
Huang X [9]	2023	Sintilimab + control / Oxaliplatin + S-1	75/75	⑦⑧	3
Wang Z [10]	2024	Sintilimab + control / Nab-paclitaxel +<br>S-1 + Trastuzumab	41/39	①②③⑤⑦⑧⑨⑩⑪⑫	7
Liu Z [11]	2024	Sintilimab + control / Docetaxel<br>+ Oxaliplatin + S-1	42/42	①②⑤⑥⑨⑪⑬	7
Wei C [12]	2022	Sintilimab + control / Docetaxel<br>+ Cisplatin + 5-Fu	43/43	①②③④⑤⑨⑪	7
Jiao F [13]	2023	Sintilimab + control / Oxaliplatin + S-1	35/79	①②⑩⑫	5
Jiang J [14]	2024	Sintilimab + control / Oxaliplatin<br>+ Capecitabine	40/40	①②④⑤⑥⑨⑪⑬	7
Li L [15]	2023	Sintilimab + control / Nab-paclitaxel	45/44	①②④⑥⑨⑩⑪⑫⑬	7
Lu X [16]	2023	Sintilimab + control / Oxaliplatin<br>+ Capecitabine	40/40	①②③⑨⑩⑪⑫	7
Hu X [17]	2024	Sintilimab + control / Oxaliplatin<br>+ Capecitabine + S-1	30/30	①②	3
Yang B [18]	2024	Sintilimab + control / Docetaxel<br>+ Oxaliplatin + S-1	52/52	①②⑤⑦⑨⑩⑫	6
Zhang S [19]	2025	Sintilimab + control / Oxaliplatin<br>+ Capecitabine	24/27	⑨⑪	3
Zhang X [20]	2025	Sintilimab + control / Oxaliplatin<br>+ Capecitabine	40/40	①②⑨⑪	5
Zhao X [21]	2025	Sintilimab + control / Oxaliplatin + S-1	41/41	②⑥⑩⑪⑬	6

### Meta-analysis of efficacy

**Figure 4:**
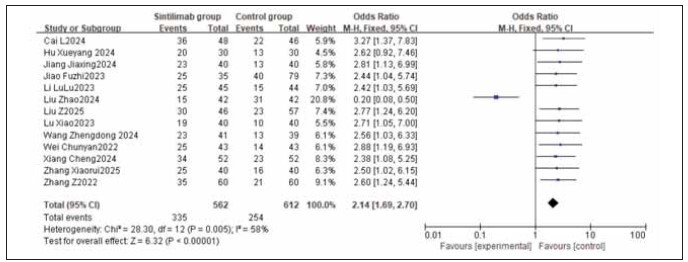
Forest plot of ORR in the meta-analysis.

**Figure 5:**
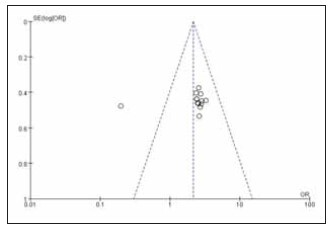
Funnel plot of the meta-analysis for ORR.

**Figure 6:**
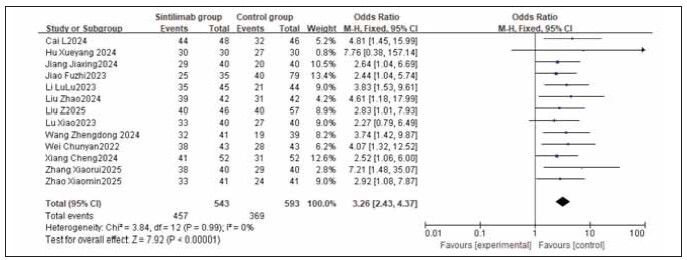
Forest plot of the meta-analysis for DRC.

**Figure 7:**
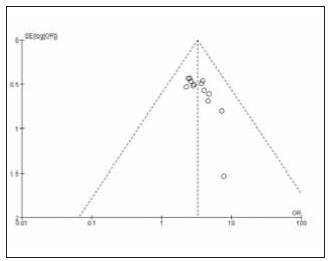
Funnel plot of the meta-analysis for DCR.

A total of 13 studies were included for both ORR (Objective Response Rate) and DCR (Disease Control Rate). Heterogeneity testing showed heterogeneity among ORR studies (I^2^ = 58.0%, P = 0.005), and homogeneity among DCR studies (I^2^ = 0.0%, P = 0.99). Random-effects and fixed-effects models were used for analysis respectively. The results indicated that both ORR and DCR were significantly higher in the treatment group compared to the control group, with the combined differences across studies being statistically significant (RR: 2.14, 95% CI: (1.69, 2.70); RR: 3.26, 95% CI: (2.43, 4.37); both P < 0.00001). It can be concluded that sintilimab improves both ORR and DCR. See Figure 4, Figure 5, Figure 6, Figure 7.

### Meta-analysis of tumor marker indicators

**Figure 8:**
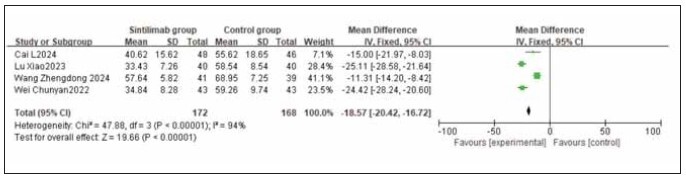
Forest plot of the meta-analysis for CA242.

**Figure 9:**
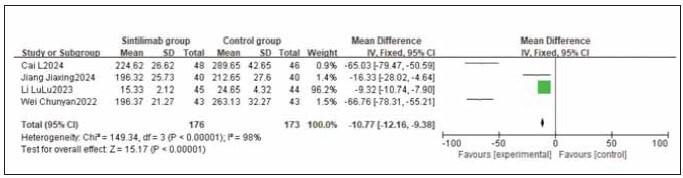
Forest plot of the meta-analysis for CA199.

**Figure 10:**
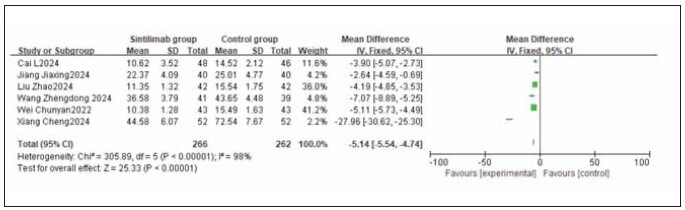
Forest plot of the meta-analysis for CEA.

A total of 4 studies for CA242, 4 studies for CA199, and 6 studies for CEA were included. Heterogeneity testing showed significant heterogeneity among the studies for CA242, CA199, and CEA (I^2^ = 94.0%, 98.0%, and 98.0%, respectively; all P < 0.05). Analysis using a random-effects model revealed that the levels of CA242, CA199, and CEA were all significantly lower in the sintilimab group compared to the control group. The combined differences across studies were statistically significant (RR: -18.57, 95% CI: (-20.42, -16.72); RR: -10.77, 95% CI: (-12.16, -9.38); RR: -5.14, 95% CI: (-5.54, -4.74); all P < 0.00001). It can be concluded that sintilimab reduces CA242, CA199, and CEA levels. See Figure 8, Figure 9, Figure 10.

### Meta-analysis of T lymphocyte subgroup indicators

**Figure 11:**
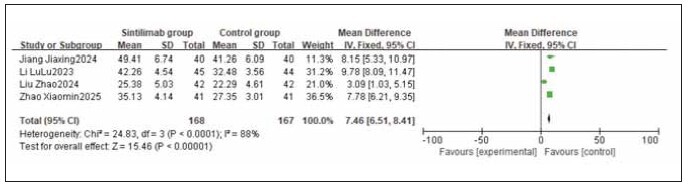
Forest plot of the meta-analysis for CD4^+^.

**Figure 12:**
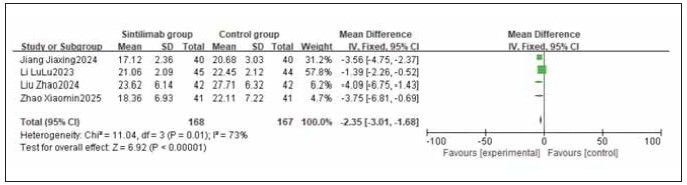
Forest plot of the meta-analysis for CD8^+^.

A total of 4 studies were included for both CD4^+^ and CD8^+^. Heterogeneity testing showed significant heterogeneity among the studies for both CD4^+^ and CD8^+^ (I^2^ = 88.0% and 73.0%, respectively; both P < 0.05). Analysis using a random-effects model revealed that CD4^+^ levels were significantly higher and CD8^+^ levels were significantly lower in the sintilimab group compared to the control group. The combined differences across studies were statistically significant (RR: 7.46, 95% CI: (6.61, 8.41); RR: -2.35, 95% CI: (-3.01, -1.68); both P < 0.00001). It can be concluded that sintilimab increases CD4^+^ and decreases CD8^+^ levels. See Figure 11, Figure 12.

### Meta-analysis of survival indicators

**Figure 13:**
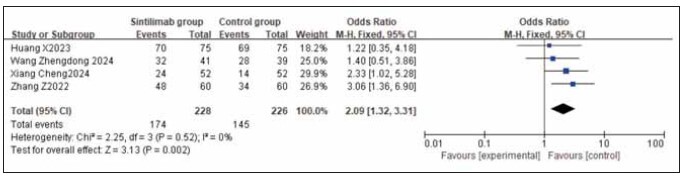
Forest plot of the meta-analysis for OS rate.

**Figure 14:**
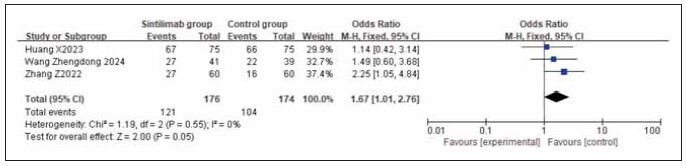
Forest plot of the meta-analysis for PFS rate.

A total of 4 studies for overall survival rate (OS) and 3 studies for progression-free survival rate (PFS) were included. Heterogeneity testing showed homogeneity among both OS and PFS studies (I^2^ = 0.0%; P = 0.52, 0.55). Analysis using a fixed-effects model revealed that both OS and PFS rates were higher in the sintilimab group compared to the control group. However, the increase in OS was statistically significant across studies (RR: 2.09, 95% CI: (1.32, 3.31), P = 0.002), while the increase in PFS was not statistically significant (RR: 1.67, 95% CI: (1.01, 2.76), P = 0.05). It can be concluded that sintilimab improves the overall survival rate. See Figure 13, Figure 14.

### Meta-analysis of adverse reaction incidence

**Figure 15:**
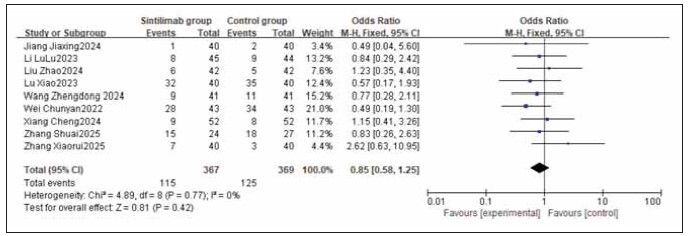
Forest plot of the meta-analysis for the incidence of myelosuppression.

**Figure 16:**
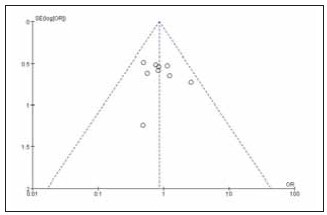
Funnel plot of the meta-analysis for the incidence of myelosuppression.

**Figure 17:**
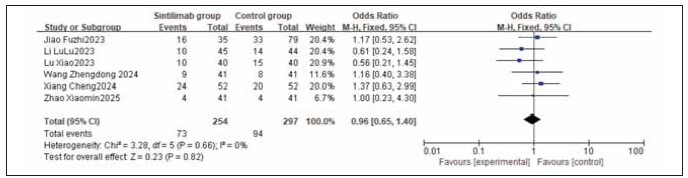
Forest plot of the meta-analysis for the incidence of nausea and vomiting.

**Figure 18:**
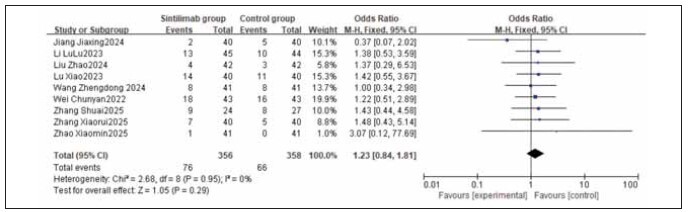
Forest plot of the meta-analysis for the incidence of liver function injury.

**Figure 19:**
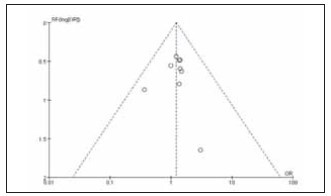
Funnel plot of the meta-analysis for the incidence of liver function injury.

**Figure 20:**
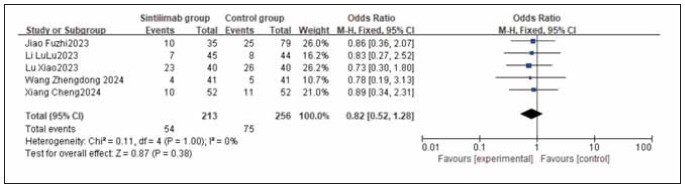
Forest plot of the meta-analysis for the incidence of peripheral sensory neuropathy.

A total of 9 studies for myelosuppression, 6 for nausea and vomiting, 9 for liver function injury, and 5 for peripheral sensory neuropathy were included. Heterogeneity testing showed homogeneity among all adverse reaction studies (I^2^ = 0.0%; P = 0.77, 0.66, 0.95, 1.00). Analysis using a fixed-effects model found that the incidences of myelosuppression, nausea and vomiting, liver function injury, and peripheral sensory neuropathy were similar between the sintilimab group and the control group. No statistically significant differences were observed across the studies (RR: 0.85, 95% CI: (0.58, 1.25); RR: 0.96, 95% CI: (0.65, 1.40); RR: 1.23, 95% CI: (0.84, 1.81); RR: 0.82, 95% CI: (0.52, 1.28); all P > 0.05). It can be concluded that sintilimab does not increase the incidence of adverse reactions. See Figure 15, Figure 16, Figure 17, Figure 18, Figure 19, Figure 20.

## Discussion

Gastric cancer is one of the most common malignant tumors of the digestive system, characterized by an insidious onset, a high propensity for metastasis, and a poor prognosis. In the early stages, treatment typically involves radical surgical interventions such as endoscopic submucosal dissection or partial gastrectomy. However, surgical procedures can trigger systemic stress responses that disrupt endocrine and metabolic pathways, ultimately hindering postoperative recovery. Furthermore, postoperative intestinal dysfunction may increase the risk of complications and further delay the restoration of physiological homeostasis [22].

In the context of laboratory medicine, tumor biomarkers such as CEA, CA242, and CA199 serve as essential indicators for disease monitoring and therapeutic response evaluation in gastrointestinal malignancies. CEA is an acidic glycoprotein widely used in colorectal cancer screening but also applicable in gastric cancer prognosis [23]. Similarly, CA242 and CA199—both carbohydrate antigens derived from colorectal cancer cell lines—are routinely quantified in clinical laboratories as part of tumor surveillance strategies. Elevated serum levels of these markers generally reflect tumor burden and disease progression [24].

This meta-analysis revealed that sintilimab administration in patients with advanced gastric cancer significantly reduced serum levels of CEA, CA199, and CA242. These reductions imply that sintilimab may exert biochemical control over tumor activity, thereby providing measurable indicators for monitoring therapeutic efficacy. Importantly, these tumor markers are accessible through standardized laboratory immunoassays such as ELISA and chemiluminescence, reinforcing the feasibility of using routine lab data to guide oncological decision-making.

In addition to biochemical markers, the study also demonstrated favorable modulation of immune-related laboratory indicators. Sintilimab significantly increased CD4 and reduced CD8 T-cell subset levels—immunological metrics commonly assessed via flow cytometry. These changes reflect restored immune function and may serve as surrogate markers of immune reconstitution following immunotherapy. As an anti-PD-1 monoclonal antibody, sintilimab blocks PD-1/PD-L1 interactions, thereby enhancing T-cell activation and reversing tumor-induced immune suppression. The resulting modulation of T-cell profiles is a critical mechanism for achieving durable anti-tumor responses.

The findings of this analysis align with the proposed mechanisms of action. Sintilimab’s high receptor occupancy rate (>95%) and affinity for PD-1 enable sustained engagement of immune checkpoints, ultimately leading to enhanced effector T-cell proliferation and macrophage activation. These immunologic shifts not only contribute to tumor regression but are also quantifiable through laboratory parameters, underscoring the value of biochemical and immunological monitoring in clinical immunotherapy [25].

Patients with malignancies often suffer from immune suppression secondary to both disease and cytotoxic treatment. Chemotherapy further exacerbates this dysfunction. By restoring immune surveillance and modulating T-cell activity, sintilimab improves systemic immunity, as evidenced by the elevation in CD4 and reduction in CD8 cell proportions. These immunological benefits support the compound’s role not only as a therapeutic agent but also as a modulator of immune health, measurable through laboratory assessment [26] [27].

From a safety perspective, this meta-analysis detected no significant increase in adverse effects such as myelosuppression, hepatotoxicity, or neurotoxicity, echoing findings from previous studies. Guo Fen et al. [28] similarly reported good tolerability and no unexpected toxicities. These results reaffirm sintilimab’s favorable safety profile, although the potential for immune-related adverse events due to overactivation of the immune system remains a clinical concern and warrants further pharmacovigilance [29] [30].

Nonetheless, certain limitations should be acknowledged. The number of eligible studies was modest, and many were published in Chinese, which may introduce publication bias and limit generalizability. Furthermore, heterogeneity (I^2^ ≥ 95%) was high in several pooled analyses, potentially confounding the robustness of conclusions. Subgroup analyses based on laboratory protocols, assay platforms, and patient immunophenotypes could help elucidate sources of variability in future work. Expanding the evidence base with high-quality, multicenter RCTs—particularly those incorporating standardized laboratory assays—would enhance the translational impact of these findings.

## Conclusion

In conclusion, sintilimab therapy in gastric cancer not only improves clinical outcomes but also significantly modulates tumor-associated and immunological biomarkers measurable in routine laboratory practice. These laboratory-based improvements under score the dual clinical and biochemical utility of sintilimab, supporting its integration into immuno - therapy regimens with measurable endpoints relevant to medical biochemistry and laboratory diagnostics.

This study has several limitations. First, noticeable heterogeneity existed in laboratory outcomes due to differences in patient populations, assay methods, and reporting standards among studies. Second, long-term adverse events and detailed subgroup analyses were rarely reported. Therefore, more high-quality, multi-center RCTs with standardized protocols are needed to strengthen our findings.

## Dodatak

### Funding

This study was supported by the 305 Hospital Independent Scientific Research Fund (No. 25ZZJJLW-008).

### Conflict of interest statement

All the authors declare that they have no conflict of interest in this work.

### Contributions

Xian Zhang and Qiang Zhao contributed equally to this work.

## References

[1] López M J, Carbajal J, Alfaro A L, Saravia L G, Zanabria D, Araujo J M, et al (2023). Characteristics of gastric cancer around the world. Crit Rev Oncol Hemat.

[2] Yin B, Dong B, Guo X, Wang C, Huo H (2022). GABPA protects against gastric cancer deterioration via negatively regulating GPX1. J Med Biochem.

[3] Mei Y, Shi M, Zhu Z, Yuan H, Yan C, Li C, et al (2022). Addition of Sintilimab to Nanoparticle Albumin-Bound Paclitaxel and S-1 as Adjuvant Therapy in Stage IIIC Gastric Cancer. Future Oncol.

[4] Wang Y, Li D (2025). Predictive value of serum tumor markers (carcinoembryonic antigen, neuron-specific enolase, and squamous cell carcinoma antigen in non-small cell lung cancer patients treated with programmed cell death protein 1 inhibitors. J Med Biochem.

[5] Wei J, Guo X, Yang X, Liu J, Duan Q, Tan Y, et al (2023). Sintilimab Plus Fluorouracil, Leucovorin, Oxaliplatin and Docetaxel Regimen as Neoadjuvant Therapy for Resectable Gastric Cancer and Biomarker Exploration. Future Oncol.

[6] Cai L, Qu L, Cheng Y, Zhang J, Li S, Wu S (2024). Study on the therapeutic effect of sintilimab combined with modified DCF regimen on advanced gastric cancer and its impact on Th1/Th2 immune balance. Anti-Cancer Drug.

[7] Liu Z, Liu A, Li M, Xiang J, Yu G, Sun P (2025). Efficacy and safety of sintilimab combined with trastuzumab and chemotherapy in HER2-positive advanced gastric or gastroesophageal junction cancer. Front Immunol.

[8] Zhang Z, Ning M Y, Han D, Li L, Li Z Z (2022). Hyperthermic Intraperitoneal Chemotherapy plus Intravenous Chemotherapy of Paclitaxel with or without Sintilimab in Gastric Cancer: A Comparative Study. J Oncol.

[9] Huang X, Fang J, Huang L, Chen H, Chen H, Chai T, et al (2023). SOX combined with sintilimab versus SOX alone in the perioperative management of locally advanced gastric cancer: a propensity score-matched analysis. Gastric Cancer.

[10] Wang Z, Pan C, Zhou A (2024). Clinical study on the combination of sintilimab and albumin-bound paclitaxel for the treatment of advanced gastric cancer patients. Chinese Journal of Clinical Pharmacology.

[11] Liu Z, Ren Y, Zhang Y (2024). The short-term efficacy of sintilimab in combination with chemotherapy for advanced gastric cancer patients and its effects on serum CEA, Tim-3, sLAG-3, and T lymphocyte subpopulations. Hebei Medical Journal.

[12] Wei C, Song L (2022). Clinical study on the combination of Xindili monoclonal antibody and modified DCF regimen for the treatment of advanced gastric cancer. Journal of Laboratory Medicine and Clinical Practice.

[13] Jiao F, Chen Y, Ji W (2023). Near-term efficacy and safety assessment of PD-1 inhibitor combined with chemotherapy as a first-line neoadjuvant therapy for locally advanced gastric adenocarcinoma. China Drug Alert.

[14] Jiang J, Feng F, Dai W (2024). Clinical efficacy of the combination of oxaliplatin and capecitabine chemotherapy with sintilimab in treating advanced gastric cancer. Clinical Military Medical Journal.

[15] Li L, Huang R, Zhang R (2023). The effects of sintilimab combined with albumin-bound paclitaxel on serum tumor marker levels and immune function in advanced gastric cancer. Clinical and Experimental Medicine Journal.

[16] Lu Xiao, Lu Z, Jin Z (2023). Efficacy and safety of sintilimab combined with chemotherapy in the treatment of advanced gastric cancer. Clinical Journal of Drug Therapy.

[17] Hu X, Li F, Wu H (2024). Efficacy and safety of sintilimab in combination with chemotherapy for stage III to IV gastric/gastric-esophageal junction adenocarcinoma. Clinical Journal of Drug Therapy.

[18] Yang B, Jiang Q (2024). The mechanism of the combination therapy of sintilimab and DOS for treating advanced gastric cancer based on KDM3A expression analysis in cancer tissues. Hebei Medical Journal.

[19] Zhang S, Liu L, Huang D (2025). Short-term efficacy and safety of cisplatin, capecitabine, and sintilimab as neoadjuvant therapy for locally advanced gastric cancer. Chinese Journal of Clinical Research.

[20] Zhang X, Shan H, Zhu Z (2025). Efficacy and safety of oxaliplatin and capecitabine in combination with sintilimab for first-line treatment of HER2-negative advanced gastric cancer. Chinese Journal of Clinical Research.

[21] Zhao X, Ding J, Wang J (2025). The Impact of Xindili Monotherapy Combined with Platinum-based Chemotherapy on Immune Indicators and Survival of Patients with Advanced Gastric Cancer. Journal of North Sichuan Medical College.

[22] Guo H, Ding P, Sun C, Yang P, Tian Y, Liu Y, et al (2022). Efficacy and safety of sintilimab plus XELOX as a neoadjuvant regimen in patients with locally advanced gastric cancer: A single-arm, open-label, phase II trial. Front Oncol.

[23] Wang R, Zhang Z, Li D, Wu N, Peng Z (2023). Association analysis of apoptosis-related gene caspase3, Integrin a subunit 1 and glutathione sulfur transferase M1 gene polymorphisms and susceptibility to gastric cardia carcinoma. J Med Biochem.

[24] Cui D, Zhou S, Wang E (2021). Application of Combined Ultrasound Endoscopy and Serum CA199, PTN, and CCSA-2 Levels in the Diagnosis of Colorectal Cancer Patients. Modern Oncology Medicine.

[25] Mei P, Long Q, Liao Y (2022). The anti-tumor mechanisms and clinical research progress of programmed death factor-1/procedural death factor ligand 1 inhibitors in neoadjuvant therapy for esophageal cancer. Chinese Journal of Experimental Surgery.

[26] Zhang L, Yue A, Duan H (2025). Efficacy of Xindili monoclonal antibody combined with TACE for the treatment of advanced primary liver cancer and its effect on T lymphocyte subpopulations. Hainan Medical Journal.

[27] Chen L, Sun P, Hu Z (2022). The effects of sintilimab combined with chemotherapy on tumor markers and immune function in advanced non-small cell lung cancer. Journal of Laboratory Medicine and Clinical Practice.

[28] Guo F, Wang Y, Shi J (2023). Clinical efficacy and safety of denizumab combined with chemotherapy for advanced gastric cancer based on real-world analysis. China Pharmaceutical.

[29] Lu Z, Wang J, Shu Y, Liu L, Kong L, Yang L, et al (2022). Sintilimab versus placebo in combination with chemotherapy as first line treatment for locally advanced or metastatic oesophageal squamous cell carcinoma (ORIENT-15): multicentre, randomised, double blind, phase 3 trial. Bmj-Brit Med J.

[30] Gao L, Tang L, Li X, Peng J, Hu Z, Liu B (2024). Efficacy and safety of sintilimab combined with apatinib as third-line or above therapy for patients with advanced or metastatic gastric cancer. Anti-Cancer Drug.

